# Incidence of multiple births in relation to current regulations in Turkey regarding embryo transfer

**DOI:** 10.1186/s12884-021-03616-9

**Published:** 2021-02-09

**Authors:** Semra Kahraman, Ipek Nur Balin Duzguner, Soner Duzguner, Yucel Sahin, Cihat Sen

**Affiliations:** 1 Istanbul Memorial Hospital, Assisted Reproductive Technologies and Reproductive Genetics Center, Piyalepasa Bulvari, Sisli, 34384 Istanbul, Turkey; 2 Istanbul Memorial Bahcelievler Hospital, Perinatal Medicine Center, 34180 Istanbul, Turkey

**Keywords:** Single blastocyst transfer, Double blastocyst transfer, Multiple pregnancy, IVF/ICSI outcome, Assisted reproductive technology (ART)

## Abstract

**Background:**

Before 2010, there were no regulations in Turkey regarding the number of embryos to be transferred in one cycle. In March 2010, regulations restricting this number were implemented by the Turkish Ministry of Health. These specify the transfer of a maximum of one embryo in the first and second cycles and a maximum of two embryos in subsequent cycles in women aged < 35, and a maximum of two embryos in women aged **≥**35 in any one cycle. Our study evaluates the effect of these regulations.

**Methods:**

This large retrospective single center study first evaluates the incidence of multiple pregnancies before and after the implementation of the 2010 regulations. Secondly, it compares the clinical outcomes of double blastocyst transfer (DBT) and single blastocyst transfer (SBT) performed in compliance with these regulations from 2014 onwards.

**Results:**

After the introduction of the 2010 regulations, the multiple pregnancy rate decreased significantly from 37.9 to 15.7%. The singleton live birth rate increased significantly, whereas multıiple live birth rates significantly decreased (*p* = < 0.001).

When the clinical outcomes of SBT and DBT performed in compliance with regulations from 2014 onwards were evaluated, in patients < 35 years, the multiple pregnancy rate decreased from 47.2% in the DBT group to 1.7% in the SBT group (*p* = < 0.001). In patients ≥35 years, in the DBT group, the twin birth rate was again high at 28.4%, whereas in the SBT group, it was only 1.8% (*p* = < 0.001). Importantly, there was no statistically significant difference in clinical pregnancy rates between these two groups.

**Conclusion:**

Turkish regulations have led to an encouragement of double embryo transfer (DET) as a routine practice, with many patients understanding it as an absolute right to have two embryos transferred. The results of our study suggest that, especially in the light of the success of blastocyst transfer, the Turkish regulations should be amended to limit the use of DET and encourage the use of single embryo transfer except in exceptional cases and particularly in women under 35 years old.

## Background

The goal of infertility treatment is to have a healthy child. However, multiple pregnancy and multiple birth, which carry an increased risk of complications in both fetuses and mothers, endanger this goal. Reducing the number of transferred embryos in ART is vital in order to reduce this risk.

Based on European Society of Human Reproduction and Embryology (ESHRE) and European IVF-monitoring Consortium (EIM) data, > 7 million ART babies were born worldwide. In 2018, De Geyter et al., reported that, in 2014, in Europe and in the USA, ART multiple birth rates were 17.5 and 22.7%, twin birth rates 17 and 22.1%, and triplet birth rates 0.5 and 0.6% respectively. The rates of twin pregnancy varied tremendously between 5.4% and more than 35% in European Countries, reflecting their differences in regulations [1].

Before 2010, there were no regulations in Turkey regarding the number of embryos to be transferred in one cycle. On 6th March 2010, regulations were announced in the Official Gazette of the Republic of Turkey [2] specifying the transfer of a maximum of one embryo in the first and second cycles and a maximum of two embryos in subsequent cycles in women < 35 years, and a maximum of two embryos in women aged **≥**35 in any one cycle. This was a very significant step, as previously there had been no limit on the number of embryos which could be transferred. Unfortunately, however, regulations have led to an encouragement of double embryo transfer (DET) as a routine practice, with many patients understanding it as an absolute right to have two embryos transferred. Furthermore, the regulations do not address the issue of embryo quality and the day of embryo transfer. Thus, clinicians are placed in a difficult position with regard to patients’ demands. For example, would it be appropriate for clinicians, under patient insistance, to agree to the transfer of two top quality blastocysts in the case of a young patient under 35 with two previous unsuccessful ART attempts? Or in the first cycle of a good responder patient, 35 or above, with many top quality blastocysts?

Our study evaluates the effect of the 2010 regulations upon the incidence of multiple births and compares the outcomes of single blastocyst transfer (SBT) versus double blastocyst transfer (DBT) performed in compliance with the regulations.

## Material and methods

### Study population

This large, retrospective single center study, based on data obtained from Istanbul Memorial Hospital, ART and Reproductive Genetics Center, evaluated single versus multiple births before and after the introduction of the 2010 Turkish regulations. The study comprised the two groups specified in the regulations as exempt from the single embryo transfer (SET) rule, namely patients < 35 years with a history of 2 or 3 unsuccessful ART attempts and patients ≥35 (35–42) years with a history of either no previous attempts or 1, 2 or 3 attempts.

The first objective of the study was to evaluate the overall effect of the 2010 regulations on multiple births, regardless of previous ART attempts and maternal age. For the period from March 2003 to June 2019, a total of 31,459 cycles with cleavage (day 2 or day 3), day 4 or blastocyst stage (day 5 or day 6) transfers were analyzed. 12,741 of these cycles were performed between January 2003 and March 2010, and 18,718 between April 2010 and July 2019.

The second objective was to evaluate the consequences of the Turkish regulations when DBT and SBT were performed in compliance with these regulations. For the period from January 2014 – July 2019, when the center’s strategy was blastocyst transfer wherever possible, a total of 4460 fresh (1569) or frozen (2891) cycles were evaluated according to two different age groups: patients < 35 with a history of 2 or 3 unsuccessful ART attempts SBT (*n* = 2288 cycles) and DBT (*n* = 585 cycles) and patients from 35 to 42 years of age with a history of either no previous attempts or 1,2 or 3 attempts. Of the 1587 cycles performed in this age group, 1114 were SBT and 473 were DBT. Females above 42 years old were not included because the significantly increased aneuploidy rate affects the implantation rate in these cases. Repeated implantation failure cases were also excluded.

In accordance with the strategy implemented in our clinic, in both groups fresh or frozen-thawed day-5 or day-6 blastocysts were transferred.

### Ovarian stimulation

For ovarian stimulation, gonadotropin-releasing hormone (GnRH) analogue suppression (short or long), GnRH antagonist protocol and recombinant follicle stimulating hormone (rFSH) (Gonal-f; Merck, Switzerland) or a combination of rFSH and recombinant luteinizing hormone (rLH) (Luveris; Merck, Switzerland) or human menopausal gonadotropin (HMG) (HMG, Ferring, Switzerland) were used. Oocyte retrievals were carried out by transvaginal ultrasound guidance 36 h after the injection of 250 mcg recombinant human chorionic gonadotropin (rhCG) (Ovitrelle; Merck, Switzerland) or GnRH analogue (Lucrin; Abbott Laboratories, USA) by transvaginal ultrasound guidance.

### Embryo scoring

Blastocysts were scored 114–120 h post-ICSI according to Gardner’s classification: excellent or top quality (TQ), good quality (GQ), moderate quality (MQ) and poor quality (PQ). Blastocysts with 3AA, 4AA, and 5AA morphology were classified as TQ blastocyst, whereas 3/4/5BB, AB or BA blastocysts were graded as GQ. The definition of moderate quality was defined as 2AA, 2AB, 2 BA blastocysts with at least 95% vitality. Blastocysts with 2BB, 2CC,3/4/5 BC, 3/4/5 CB, morula or below 90% vitality for all blastocysts were graded as poor quality [3].

Serum β-hCG was measured 9 days after blastocyst transfer. The implantation rate was calculated as the number of gestational sacs observed at ultrasonography screening divided by the number of embryos transferred. When pregnancy occurred, the same daily doses of progesterone vaginal gel were continued until the 10th week of gestation. A transvaginal ultrasound was performed at 7 weeks in order to monitor early pregnancy, with the presence of a fetal heart beat indicating vital pregnancy. Ongoing pregnancy was defined as a 12-week viable pregnancy.

### Embryo vitrification and thawing

Good or top-quality blastocysts were vitrified either on day 5 or day 6 using Kitazato vitrification media and cryotops® as carriers. Blastocysts were thawed with Kitazato warming media. 30 min later, embryos were checked for survival. 2 h after warming, embryos were re-checked for re-expansion. Before embryo transfer (3 to 3.5 h after thawing), they were checked once again for hatching, extensive cytoplasmic granulation and the presence of necrotic foci, the predictors of the rates of implantation, pregnancy, and live births [4]. Embryos that had > 50% arrested/necrotic TE or ICM cells were classified as not surviving. Only vital blastocysts with at least 80% re-expansion were transferred.

## Statistical analysis

Statistical analysis was performed with Statistical Package for Social Sciences (SPSS) Mac version 21 (SPSS INC. CHİCAGO, IL, USA). Descriptive statistics and categorical variables are given as numbers and percentages. Numerical variables are given as mean ± standard deviation. The minimum and maximum values were included in the calculation. The distribution of continuous variables was assumed normal because the sample size was large enough to do so. Independent Two Samples t-Test was used to compare data. Nominal variables were analyzed with Pearson’s Chi-Square Test and Fisher’s Chi-Square Test. *p* value of < 0.05 was considered as statistically significant for all tests at 95% confidence interval. The relative change is calculated by subtracting the end value from the initial value, dividing the result by the initial value and multiplying by one hundred. The result is expressed in percentage terms.

## Results

Results related to the first aim of the study, the evaluation of the incidence of multiple pregnancies before and after 2010, are shown in Tables 1 and 2. Demographic and ART cycle characteristics of patients are shown in Table 1. Statistically significant differences were observed in the female age, number of previous cycles and duration of infertility. The female age was older, the number of previous cycles was higher and the duration of infertility was shorter. The mean number of aspirated oocytes and fertilizied oocytes with 2PN were significantly different before and after the 2010 regulations**.** No differences were observed in the cause of infertility and in the mean number of MII oocytes**.** Among infertility causes, the number of cases with diminished ovarian reserve (DOR) and cases with recurrent spontaneous pregnancy losses (RPL) were significantly higher in the pre-2010 group. Cases with ovulatory dysfunction and cases with tubal factor were both found to be significantly lower in the post-2010 group.

**Table 1:** Patient demographics, duration of and reasons for infertility and cycle characteristics before and after regulations

	2003-April 2010 ***n*** = 12,741	April 2010–July 2019 ***n*** = 18,718	***P***-value
Age, years, mean ± SD	32.63 ± 5.55	33.24 ± 5.37	^a^ < 0.001*
Maternal BMI (kg/m^2^), mean ± SD	25.48 ± 4.54	25.04 ± 4.53	^a^ < 0.001*
Previous cycle number, mean ± SD	2.29 ± 1.69	3.48 ± 2.69	^a^ < 0.001*
Duration of infertility, years, mean ± SD	7.59 ± 5.38	5.74 ± 4.54	^a^ < 0.001*
Infertility cause, n (%)			
Male Factor	4399 (34.5)	6219 (33.2)	^b^0.016*
Diminished ovarian reserve	1742 (13.6)	3478 (18.5)	^b^ < 0.001*
Endometriosis	424 (3.3)	734 (3.9)	^b^0.006*
Ovulatory dysfunction	1073 (8.4)	1057 (5.6)	^b^ < 0.001*
Genetics	652 (5.1)	875 (4.7)	^b^0.08
Recurrent spontaneous abortion	321 (2.5)	839 (4.5)	^b^ < 0.001*
Tubal Factor	1796 (14.0)	2301 (12.3)	^b^ < 0.001*
Unexplained infertility	2334 (18.3)	3215 (17.2)	^b^0.01*
Number of aspirated oocytes, mean ± SD	12.90 ± 7.81	11.62 ± 8.63	^a^ < 0.001*
MII, mean ± SD	9.39 ± 5.98	9.33 ± 7.03	^a^0.406
PN2, mean ± SD	7.37 ± 4.48	7.81 ± 6.01	^a^ < 0.001*
Number of embryos transferred, n (%)			
1	1803 (14.1)	10,600 (56.6)	
2	4105 (32.2)	8120 (43.4)	
3	4025 (31.6)	–	
4	1916 (15.0)	–	
5	882 (6.8)	–	
Embryo transfer day, n (%)			
D2–3	5977 (46.9)	2647 (14.1)	
D4	3448 (27.0)	2225 (11.9)	
D5–6	3316 (26.1)	13,846 (74)	

*BMI* Body mass index, *MII* Maturated oocytes, *PN2* Normally fertilized oocytes

^a^Independent Two Samples t-Test ^b^Chi-Square Test *p < 0,05

**Table 2:** Multiple pregnancy and live birth rates before and after March 2010 regulations

	2003-April 2010 ***n*** = 12,741	April 2010–July 2019 ***n*** = 18,718	***P***-value
Multiple pregnancy	1992 (37.9)	1683 (15.7)	^a^ < 0.001*
Live Birth n (%)	4148 (32.5)	8472 (45.2)	^a^0.02*
Singleton Live Birth	2719 (65.5)	7119 (84)	^a^ < 0.001*
Multiple Live Birth	1429 (34.5)	1353 (16)	^a^ < 0.001*
Twin Live Birth	1346 (32.5)	1320 (15.6)	^a^ < 0.001*
Triplet Live Birth	78 (1.9)	32 (0.3)	^a^ < 0.001*
Quadruplet Live Birth	3 (0.0)	1 (0.0)	N/A
Term Live Birth, n (%)	3157 (76.1)	6753 (79.7)	^a^ < 0.001*
Preterm Live Birth, n (%)	989 (23.8)	1719 (20.2)	^a^ < 0.001*

^a^ Chi-Squared Test *p < 0,05

With the implementation of regulations, the percentage of SET increased significantly. There was also a significant increase in the percentage of DET, most probably as a result of the prohibition of the transfer of three or more embryos and therefore more patients requesting DET within the regulations. Furthermore, the percentage of patients with embryo transfer on day-2 or day-3 of embryo development decreased dramatically from 46.9% before 2010 regulations to 14.1% after their introduction. Similarly, there was a significant decrease in day-4 transfer from 27 to 11.9%. Conversely, the percentage of cases with day 5 to day 6 transfer increased significantly from 26.1 to 74%. This reflects the change in our center protocol from cleavage stage to blastocyst stage in order to improve the selection process for SET.

Table 2 shows multiple pregnancy and live birth rates before and after March 2010 regulations. The multiple pregnancy and multiple live birth rates decreased significantly from 37.9 to 15.7% and from 34.5 to 16% respectively. Live birth rates increased significantly after the regulations (*p* = 0.02). The singleton live birth rate increased significantly, whereas twin and triplet live birth rates significantly decreased. The rate of preterm births significantly decreased. These were all positive developments. However, despite a significant rise in SET, the incidence of twin births remained undesirably high at 15.5%.

The second part of our study is based on cases of single or double blastocyst transfer and evaluates the consequences of the regulations in the two groups specified in the regulations as exempt from the single embryo transfer rule.

In Table 3, patients under 35 years old with 2 or 3 unsuccessful previous cycles with single or double day-5 or day-6 blastocyst transfer are compared. The female age, AMH level and the mean number of MII oocytes were significantly different between the two groups. No significant differences were observed in terms of BMI, mean number of aspirated oocytes and fertilized oocytes, biochemical and clinical pregnancies, clinical pregnancy losses and live birth rates. The main differences observed between the two groups were the rates of implantation, preterm live births, single and twin live births. The implantation rate in women under 35 years old was 68.7% with SBT, whereas this rate was 51.3% with DBT. A significantly higher preterm birth rate was observed in DBT cases compared to SBT cases. A significantly higher single live birth rate was observed in SBT compared to DBT. Twin live birth rates were significantly lower in SBT compared to DBT. There were no triplet live births in SBT and the percentage of triplet live births was 0.86% in DBT.

**Table 3:** Comparison of single and double blastocyst embryo transfer cycle results between January 2014 – July 2019 in women under 35 years of age with 2 or 3 previously, unsuccessful ART treatments

	SBT ***n*** = 2288	DBT ***n*** = 585	***P***-value
Age years, mean ± SD	29.31 ± 3.42	29.7 ± 3.20	^a^ < 0,05*
AMH, (ng/ml), mean ± SD	4.03 ± 3.38	3.50 ± 3.38	^a^ < 0.01*
Maternal BMI, (kg/m^2^), mean ± SD	24.43 ± 4.48	24.33 ± 4.15	^a^0.646
Number of aspirated oocytes, mean ± SD	15.17 ± 9.08	14.62 ± 8.19	^a^0.157
MII, mean ± SD	12.51 ± 7.52	11.76 ± 6.50	^a^ < 0,05*
PN2, mean ± SD	10.33 ± 6.53	9.89 ± 5.71	^a^0.111
Implantation Rate, (%)	68.7	51.3	^a^ < 0.001*
Biochemical Pregnancy, n (%)	1705 (74.5)	436 (74.5)	^b^0.81
Clinical Pregnancy, n (%)	1542 (67.4)	395 (67.5)	^b^0.85
Biochemical Abortion, n (%)	163 (9.5)	41 (9.4)	^b^0.84
Clinical Abortion n (%)	198 (12.8)	42 (10.6)	^b^0.69
Total Live Birth, n (%)	1319 (57.6)	347 (59.3)	^b^0.49
Singleton Live Birth, n (%)	1296 (98.2)	180 (51.8)	^b^ < 0.001*
Twin Live Birth, n (%)	23 (1.7)	164 (47.2)	^b^ < 0.001*
Triplet Live Birth, n (%)	–	3 (0.86)	N/A
Term Live Birth, n (%)	1168 (88.6)	226 (65.1)	^b^ < 0.001*
Preterm Live Birth, n (%)	151 (11.4)	121 (34.9)	^b^ < 0.001*

*SBT* Single blastocyst transfer, *DBT* Double blastocyst transfer, *AMH* Anti-Mullerian Hormone, *BMI* Body mass index

^a^Independent Two Samples t-Test ^b^Chi-Squared Test *p < 0,05

Table 4 shows a comparison of single and double blastocyst embryo transfer cycle results in women between 35 and 42 years of age with a history of either no previous attempts or 1, 2 or 3 attempts. The mean female age, the mean number of MII oocytes and the mean number of fertilized oocytes were significantly different between the two groups. No significant difference was observed in patients between the age of 35 and 42 with a history of either no previous attempts or 1, 2 or 3 attempts in terms of BMI, AMH level and mean number of aspirated oocytes. The implantation rate in women between 35 and 42 years of age was 59.6% with SBT and 36.2% with DBT. The clinical miscarriage rate was significantly lower in the SBT group. The preterm birth rate was significantly higher in DBT group compared to SBT cases. The live birth rate was significantly higher in the SBT group. The singleton live birth rate was significantly higher in the SBT group. A dramatically higher twin birth rate was observed in DBT cases compared to SBT cases.

**Table 4:** Comparison of single and double blastocyst embryo transfer cycle results between January 2014 – July 2019 in women between 35 and 42 years of age with a history of either no previous attempts or 1, 2 or 3 attempts

	SBT ***n*** = 1114	DBT ***n*** = 473	***P***-value
Age years, mean ± SD	37.32 ± 2.02	37.60 ± 2.11	^a^ < 0.05*
AMH, (ng/ml), mean ± SD	2.16 ± 2.28	1.93 ± 2.14	^a^0.09
BMI, (kg/m^2^), mean ± SD	25.07 ± 4.79	25.38 ± 4.70	^a^0.236
Number of aspirated oocytes, mean ± SD	10.89 ± 8.29	10.14 ± 7.27	^a^0.074
MII, mean ± SD	9.19 ± 6.92	8.31 ± 5.66	^a^ < 0.01*
PN2, mean ± SD	7.59 ± 5.85	6.98 ± 4.77	^a^ < 0.05*
Implantation Rate, (%)	59.6	36.2	^a^ < 0.001*
Biochemical Pregnancy, n (%)	718 (64.4)	291 (61.5)	^b^0.25
Clinical Pregnancy, n (%)	659 (59.1)	256 (54.1)	^b^0.055
Biochemical losses, n (%)	59 (8.2)	35 (12)	^b^0.045*
Clinical Miscarriage n (%)	76 (11.5)	56 (21.9)	^b^ < 0.001*
Total Live Birth, n (%)	569 (51)	194 (41)	^b^ < 0.001*
Singleton Live Birth, n (%)	559 (98.2)	138 (71.1)	^b^ < 0.001*
Twin Live Birth, n (%)	10 (1.8)	55 (28.4)	^b^ < 0.001*
Triplet Live Birth, n (%)	–	1 (0.4)	N/A
Term Live Birth, n (%)	491 (86.3)	129 (66.5)	^b^ < 0.001*
Preterm Live Birth, n (%)	78 (13.7)	65 (33.5)	^b^ < 0.001*

*SET* Single blastocyst transfer, *DET* Double blastocyst transfer, *AMH* Anti-Mullerian Hormone, *BMI* Body mass index

^a^Independent Two Samples t-Test ^b^Chi-Squared Test *p < 0,05

**Fig. 1:**
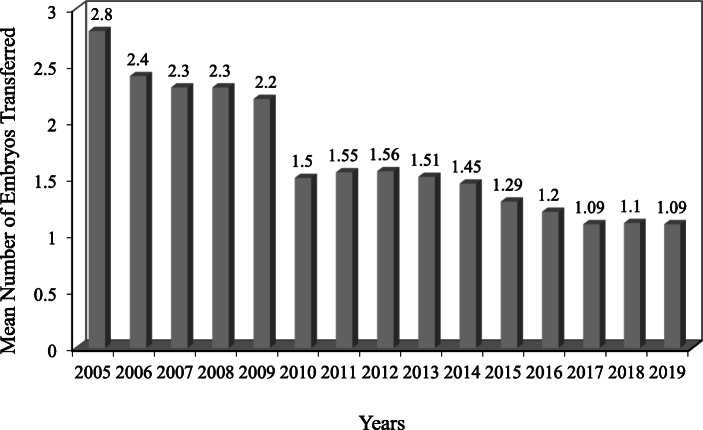
The mean number of embryos transferred per cycle over the period 2005 to 2019 in young patients below the age of 35

Figure 1 shows the mean number of embryos transferred per cycle over the period 2005 to 2019 in young patients below the age of 35. It declined from 2.8 in 2005 to 1.09 in 2019. There was a fall in 2010 with the introduction of regulations, when we allowed DET upon patient request in compliance with the regulations regarding number of previous cycles and patient’s age. However, there was a further, more dramatic fall when we changed the strategy at our clinic in 2017. From 2017 onwards, SBT has been performed for the vast majority of our patients regardless of their age and the number of previous cycles. Over the period from 2005 to 2019, there was a 61% (2.56 times) relative decrease in the mean number of embryos transferred.

**Fig. 2:**
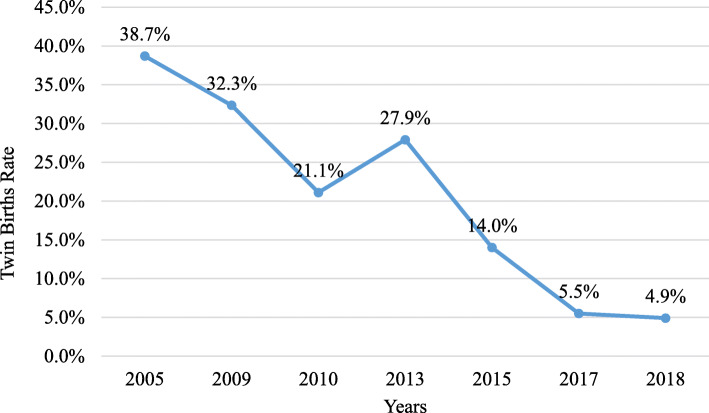
The twin birth rates in Istanbul Memorial Hospital by years

For the calculation of the relative change we analyzed the difference between the number of embryos transferred in Fig. 1 and the twin births rate in Fig. 2 from year to year using relative change calculation.

Figure 2 shows the twin birth rates in Istanbul Memorial Hospital by years. There has been a dramatic decline in the twin birth rate over the past 13 years from 38.7 to 4.9%, without affecting the pregnancy rate. This represents an 87.3% (7.99 times) relative decrease.

## Discussion

Multiple pregnancies must be regarded as a serious complication of ART cycles, resulting in medical, psychological, social and financial problems. Although high order multiple pregnancies are rare in countries where there are regulations limiting the number of embryos which can be transferred, twin pregnancies remain a common problem in ART. In a study by Santana et al., 2016, twin pregnancy maternal complications were compared to singleton pregnancies. Potentially maternal life-threatening conditions were found to be significantly higher in the twin deliveries group. Furthermore, the risk of early term birth, at 39.2%, was significantly higher in twins [5]. If the preterm risk was added (53.8%), twins had over a 90% risk of being born before 39 weeks and were therefore at an increased risk of adverse outcomes such as neurodevelopmental disability, including cerebral palsy (CP) [6, 7]. A cohort study by Goldsmith et al., 2018, concluded that a higher risk of CP in children born after ART is strongly associated with the high proportion of multiple pregnancies and preterm deliveries in these pregnancies [8]. Thus, it is vital that all countries should have regulations aimed at reducing not only the incidence of high order multiple pregnancies but also of twin pregnancies [9]. Van Landuyt et al. compared the IVF outcome before and after the Belgian embryo transfer policy. The multiple pregnancy rate was reduced from 29.1 to 9.5% (all patients) and from 28.9 to 6.2% in women < 36 years. Most twins were observed in the third cycle of patients < 36 years and in the first three cycles in women of 36–39 years. Clinical pregnancy rates were not compromised by the new law [10].

The first aim of our study was, therefore, to evaluate the incidence of multiple pregnancies before and after 2010, when regulations regarding the numbers of embryos to be transferred in one cycle were introduced for the first time in Turkey. Information regarding the incidence of multiple births since the introduction of these regulations is not available from the Ministry of Health. Our study was therefore designed as a retrospective single center study of 31,459 cycles, the highest number of cycles carried out in a single center in Turkey between the years of 2003 and 2019. The data includes all patients, regardless of age (between 17 and 46 years) and regardless of the stage of embryo transfer (day 2,3,4,5 and 6) and the number of embryos transferred. The data from our center for the period from 2014 to 2019, when a maximum of two blastocysts were transferred, excludes patients of over 42 years. After the 2010 regulations, the percentage of patients with single embryo transfer cycle increased dramatically from 14.1% before regulations to 56.6% between 2010 and 2019. As can be seen in our study, the 2010 regulations were a positive development. However, despite a significant rise in SET and despite a fall in the incidence of multiple pregnancies from 34.2 to 15.5%, the latter was still undesirably high. Furthermore, regulations do not address the issue of the day of the embryo transfer. Should it be day 2 transfer, day 3, day 4 day 5 or later?

The second part of our study compares SET and DET at blastocyst stage in the two groups specified in the regulations as exempt from the single embryo transfer rule and covers the period from January 2014 – July 2019. When only blastocyst stage embryos were transferred, the twin birth rates in both the < 35 years group and the ≥35 (35–42) group were extremely high when DBT was used (47.2, 28.4% respectively) as compared to SBT (1.7, 1.8% respectively). The most dramatic decline in the number of embryos transferred occurred in 2017 with the implementation of our SBT strategy in younger age groups, when the mean number of blastocysts transferred fell from 1.5 to 1.09. We saw a dramatic decline in the twin birth rate from 38.7% in 2005 to 4.9% in 2018 with no effect on the pregnancy rate (Fig. 2). The main reason for this has been our shift in strategy to SBT regardless of patient age and number of previous cycles. Importantly, there was no statistically significant difference in clinical pregnancy rates between these two groups (67.4% vs 67.5%). According to our results, the failure of two previous cycles does not justify the transfer of two blastocysts in cases under 35. However, as a result of the wording of the regulations, although all such patients were strongly advised to have SBT, many nevertheless demanded DBT. Thus, in practice, these regulations run the risk of increasing the incidence of multiple births in this age group with no advantage in terms of clinical pregnancy rates.

Similarly, many patients in the ≥35 years age group understand the 2010 regulations as an absolute right to have two embryos transferred. Unfortunately, this aspect of the regulations encourages double embryo transfer as a routine procedure instead of single embryo transfer in this age group, in whom DBT resulted in a high twin birth rate at 28.4%, whereas in the single blastocyst transfer group it was only 1.8%. Again, there was no statistically significant difference in clinical pregnancy rates between the SBT and DBT groups. These findings show that DBT between the ages of 35 and 42 does not increase the chance of pregnancy, while it greatly increases the risk of multiple births. Clearly, DBT was not the best strategy for patients up to the age of 40 with between zero and three previous, unsuccessful cycles. For older patients between the ages of 38–42 with multiple unsuccessful attempts who feel increasingly anxious about the chances of success, and who therefore request DBT, the alternative of selecting one euploid embryo for SBT may be advised [11]. Further studies focusing on these older patients are needed to determine in which age groups and in which individual cases DBT might be a better alternative.

The transfer of a good-quality embryo is a key factor in IVF success. Over the past 15 years, with dramatic improvements in ART, we have steadily shifted our strategy from cleavage stage towards single blastocyst transfer in good responder patients, regardless of patient age and number of previous cycles. Extended culture to the blastocyst stage has been improved through the use of better culture media and through quality controls and quality assessments. A dramatic improvement in cryopreservation has been achieved through the introduction of the vitrification technique and because of extended culture to the blastocyst stage, the availibility of trophectoderm biopsy has become possible. This further offers improved accuracy of diagnosis and assessment of entire chromosomal complement, particularly in advanced maternal age cases.

Our study has the limitation of being retrospective design using data from a single center, rather than from Turkey as a whole. However, the high number of cycles evaluated enable us to draw clear conclusions applicable to the country as a whole. It shows that, when using a blastocyst stage transfer strategy, this regulation can result in a twin birth rate of over 47.2% in young patients, whereas this can be reduced to 1.7% with SBT and that, similarly, in older patients, DBT can result in a twin birth rate of over 28%, which can be reduced to only 1.8% with SBT.

## Conclusions

Although the 2010 Turkish regulations restricting the number of embryos to be transferred in one cycle was a positive step, nevertheless they allow DET both in young patients below 35 years old with two unsuccessful previous attempts and in patients ≥35 in any one cycle. Thus, the risk of multiple pregnancy continues. The results of our study suggest that, especially in the light of the success of blastocyst transfer, the Turkish regulations should be amended in order to limit the use of DET and encourage the use of SET, apart from in specified, exceptional cases and particularly in women under 35 years old.

## Data Availability

The datasets generated and/or analyzed during the current study are not publicly available due to patient privacy and hospital policy but are available from the corresponding author on reasonable request.

## Abbreviations

ART: Assisted Reproductive Technology
ESHRE: European Society of Human Reproduction and Embryology;
EIM: European IVF-monitoring Consortium
SET: Single embryo transfer
DET: Double embryo transfer
SBT: Single blastocyst transfer
DBT: Double blastocyst transfer
GnRH: Gonadotropin-releasing hormone
rFSH: Recombinant follicle stimulating hormone
rLH: Recombinant luteinizing hormone
HMG: Human menopausal gonadotropin
rhCG: Recombinant human chorionic gonadotropin
TQ: Excellent quality.
GQ: Good quality
MQ: Moderate quality
PQ: Poor quality
SPSS: Statistical Package for Social Sciences
BMI: Body mass index
MII: Maturated oocytes
PN2: Normally fertilized oocytes
AMH: Anti-Mullerian Hormone
CP: Cerebral palsy
